# Malaria and haematologic parameters of pupils at different altitudes along the slope of Mount Cameroon: a cross-sectional study

**DOI:** 10.1186/1475-2875-12-193

**Published:** 2013-06-09

**Authors:** Helen K Kimbi, Irene UN Sumbele, Malaika Nweboh, Judith K Anchang-Kimbi, Emmaculate Lum, Yannick Nana, Lucy M Ndip, Henry Njom, Leopold G Lehman

**Affiliations:** 1 Department of Zoology and Animal Physiology, Faculty of Science, University of Buea, P.O. Box 63, Buea, SWR, Cameroon; 2 Department of Microbiology and Parasitology, Faculty of Science, University of Buea, P.O. Box 63, Buea, SWR, Cameroon; 3 Emerging infectious Disease Laboratory, Faculty of Science, University of Buea, P.O. Box 63, Buea, SWR, Cameroon; 4 Department of Animal Biology, Faculty of Science, University of Douala, P.O. Box 2701, Douala, Cameroon

**Keywords:** Malaria, Prevalence, Altitude, Pupils, Haematologic Profile, White Blood Cell Count

## Abstract

**Background:**

Haematologic abnormalities are features in *Plasmodium falciparum* infection, and anaemia is an inevitable outcome. This study examines the influence of malaria status and altitude on haematologic parameters in school-aged pupils.

**Methods:**

A cross-sectional study was conducted among 728 school pupils aged between four and 15 years at three different altitudinal ranges along the slope of the Mount Cameroon region. The investigative methods included the use of questionnaire, clinical evaluation and laboratory investigations. Blood sample collected from each child was used for the preparation of blood films for detection of malaria parasites and assessment of malaria parasite density as well as full blood count determination using an automated haematology analyzer.

**Results:**

The prevalence of malaria in the study population was 33.8% and 64.2% (158/246) of these were asymptomatic (AM). Pupils in lowlands had a significantly (P <0.05) prevalence (95% confidence interval, CI) of malaria (60.6%, CI = 54.6–65.9%) than those in middle belt (29.1%, CI = 23.9–34.8%) and highlands (7.7%, CI = 6.1–9.8%), while those in middle belt had significantly higher geometric mean parasite density (475) than those in lowlands (233) and highlands (388). The prevalence of malaria was significantly higher in children that presented with fever (40.4%, CI = 33.8–47.2%) when compared with afebrile subjects (31%, CI = 27–35.2%). Pupils with AM had a higher prevalence of leucopaenia (43.7%, CI = 35.8–51.8%), microcytosis (27.2%, CI = 20.5–34.9%), hypochromasia (27.8%, CI = 21–35.5%) and thrombocytopaenia (14.9%, CI = 8.9–22.8%) when compared with those with clinical malaria (CM). All mean haematological parameters were comparable in pupils with CM and AM, except for the mean white blood cell (WBC) counts. Pupils with AM had significantly lower (P = 0.02) mean WBC counts (5.1 ± 2.5 × 10^9^/L) than those with CM (5.9 ± 2.3 × 10^9^/L). Age, altitude and malaria parasitaemia was of significant influence on several haematological parameters.

**Conclusion:**

Altitude influenced the distribution and density of malaria parasites and was of confounding influence on the haematologic profiles. These results highlight the insidious effects of AM on the haematologic components.

## Background

Malaria still remains a major public health problem in sub-Saharan Africa in spite of an increase in control measures. In Cameroon it is estimated that 41% of the population has at least one episode of malaria each year with children under five years and pregnant women mostly affected [1]. The clinical consequence of an infection in a child depends on many factors, which are often ill-defined and determine the outcome in each child. The clinical effect of a malarial infection in an African child depends on parasite, host, geographical and social factors. These converge in the child to result in a range of outcomes, from an asymptomatic infection to severe disease and death [2].

Although fever usually has a high sensitivity but poor specificity for the diagnosis of malaria, it still serves as the entry point for presumptive treatment of malaria in African children who, if left untreated, run the risk of severe complications and even death. The presumptive treatment of all fevers is the most risk-adverse approach to managing "malaria" across Africa [3].

Haematologic abnormalities are considered a feature in *Plasmodium falciparum* infection. The severity of haematologic disease caused by *Plasmodium* is related to the ability of the parasites to invade and grow in different red cell populations as well as the intrinsic growth rate of the parasite [4]. Anaemia is consequently an inevitable outcome of malaria infection, but the haematocrit (Hct) in some patients with high parasite densities does not decrease as much as would be expected from the destruction of the parasitized red blood cells [5]. Similar to haemoglobin (Hb) levels, haematocrit values vary according to age, gender, and altitude. Lower Hb concentration has been associated with malaria parasitaemia in young children [6-9], symptomatic malaria cases and persons with patent parasitaemia [10,11] than in malaria negative subjects. However, the impact of asymptomatic malaria (AM) on haematological parameters in school-aged children is unclear.

Changes in leucocyte and platelet counts are present in malaria. Leucocytosis has been associated with severe disease [12] while thrombocytopaenia has been identified as a key indicator of malaria in febrile patients [11]. Although thrombocytopaenia is a recognized complication of falciparum malaria in adults, very little is known about platelets in children with asymptomatic and mild malaria. This study examines the influence of malaria status and altitude on haematologic parameters in school aged pupils. Specifically, the haematologic parameters of malarial parasite positive pupils were compared with those negative as well as pupils with clinical malaria (CM) and those with AM at different altitudes.

## Methods

### Study sites and subjects

The study was carried out in primary school children of both sexes aged four to 15 years at three different altitudinal ranges along the slope of the Mount Cameroon region. The sites were classified as lowlands (0-167 m above sea level (asl)), middle belt (600-650 m asl) and highlands (897-918 m asl). In lowlands, a total of 303 pupils were recruited into the study from Catholic school (CS) Bota, CS Gardens and CS Ngeme. In middle belt, 141 pupils were recruited from Government school (GS) Molyko while in highlands, the 284 pupils who participated in the study were recruited from GS Bova, GS Bonakanda and Jamandiale primary school. Pupils were enrolled into the study only if they were pupils in one of the chosen schools, presented a signed consent form from parent/legal guardian, and succumbed to the blood collection procedure.

The temperatures in lowlands (Limbe) are high and fairly constant ranging between 25°C and 30°C on average [13] with abundant rainfall ranging from 5,500 to 6,500 mm per annum. In both middle belt and highlands (Buea), weather records from the Cameroon Development Corporation indicate a mean relative humidity of 80%, an average rainfall of 4,000 mm and a temperature range of 18-27°C. In all three altitudes there are two distinct seasons: a cold rainy season which spans from mid-March to October and a warm dry season with frequent light showers which runs from November to February. Malaria is endemic in the Mount Cameroon region with an average (44) of one to 100 malaria cases per 1,000 per year [14]. Transmission occurs all year round with two peaks in season, the first in April and May and the second in October and November. These correspond with the beginning and end of the rainy season respectively. *Plasmodium falciparum* is the main species and *Anopheles gambiae* is the main vector species [15].

### Study design

This cross-sectional study was carried out between the months of May and November, 2011 to coincide with the peak of malaria transmission season. In each school, a sensitization rally was organized with the teachers of the schools to explain the purpose and benefits of the study before the sampling was done. Informed assent forms were sent to parents/guardians via the children stating the purpose of the study as well as the benefits and the amount of blood that had to be collected from each child. Only children who brought back signed assent forms were included in the study. The investigative methods included the use of questionnaire to assess socio economic status (SES), clinical evaluation and laboratory investigation.

### Questionnaire

A simple structured questionnaire was administered to pupils to obtain data on key socio-economic variables including the parent/guardian’s assets and the use of mosquito control measures. Based on these, the socio-economic status (SES) was classified as poor (those living in plank houses, have no TV/radio, no car, use firewood kitchen and pit toilets), middle class (those living in block houses, have flush toilets, TV/radio, firewood/gas kitchen but no car) and rich (those having all what the middle class children had and a car).

### Clinical evaluation

Data on each child’s name, sex and age was obtained from the school register. The axillary temperature of each child was measured using a clinical thermometer. A pupil was considered febrile when he/she had a body temperature ≥37.5°C.

### Laboratory procedure

Approximately 2 mL of blood was collected from each child by venipuncture into a 2 mL sterile disposable syringe (Cathy Yougo) and dispensed into ethylene-diamine-tetra-acetate (EDTA) tubes. Drops of whole blood were dispensed immediately on slides for the preparation of thick and thin blood films for detection and speciation of malaria parasite as described by Cheesbrough [16]. The blood samples in EDTA tubes were transported on ice blocks to the Emerging Infectious Diseases Laboratory, University of Buea for storage at 4°C. The blood samples were used for the assessment of malaria parasite density as well as full blood count.

Parasite density per μL of blood was determined on the basis of number of parasites per 200 leukocytes on thick blood film with reference to subjects’ white blood cell counts (WBC). Slides were considered positive when asexual forms and/or gametocytes of any *Plasmodium* species were observed on the blood film [16]. Slides were read by two independent parasitologists and in the case of any disparity they were read again by a third person.

### Haematology

The tubes containing the anticoagulated blood were rocked gently on a multitube rotator. The complete blood count: red blood cell (RBC), WBC, Hb, Hct, platelet, mean corpuscular volume (MCV), mean corpuscular haemoglobin (MCH), and mean corpuscular haemoglobin concentration (MCHC) was run following the manufacturer’s instructions using an automated haematology analyzer, the Beckman Coulter counter (URIT 3000).

### Definitions and end points

• Asymptomatic malaria was defined as the presence of *Plasmodium* with an axillary temperature of <37.5°C;

• Clinical malaria was defined as the presence of any species of *Plasmodium*, with an axillary temperature of ≥37.5°C;

• Parasitaemia was categorized as low (<1,000 parasite/μL blood), moderate (1,000-4,999 parasites/μl blood) and high (≥5,000 parasites/μL blood;

• A haemoglobin level of <11g/dL was classified as anaemic;

• Microcytosis was defined as MCV of less than 73 fl [17];

• Hypochromasia was defined as a MCHC of less than 320 g/L [18];

• Leucopaenia was defined as WBC < .4.5 × 10^9^/L;

• Thrombocytopaenia was defined as platelet count <150,000/μL.

### Statistical analyses

Data was entered into spread sheets using Microsoft Excel and analysed with the statistical package for social sciences (SPSS) version 17 (SPSS, Inc, Chicago, IL, USA) and Epi info version 7 (CDC). Data was summarized into means and standard deviations, and percentages were used in the evaluation of the descriptive statistics. Proportions were compared using the Chi-square test (χ^2^). Means ± standard deviations (SD) were compared using independent sample t-test, Mann-Whitney U test and analysis of variance (ANOVA) where appropriate. Exploratory analyses were performed to select and prioritize potential confounders of haematological values to be entered into a multiple linear regression (MLR) model. Any potential confounder that had at least a modest (P <0.2) relation with both the dependent variable and the confounder of interest was included in the later MLR models. Malaria parasite counts were log transformed before analysis and entered into the model. Significant levels were measured at 95% confidence level (CI) with significant differences recorded at P <0.05.

### Ethical considerations

Before commencement of the study, an ethical clearance was obtained from the South West Regional Delegation of Public Health while administrative clearances were obtained from the Regional Delegation of Basic Education as well as from the Catholic Education Board. Pupils participated in the study if a parent or guardian signed the informed assent form. The parents or guardians and their children were informed that their participation in the study was voluntary and they could withdraw at any time without any explanation.

## Results

### Baseline characteristics of the study population

Of the 728 pupils with a mean age of 8.62 ± 2.23 (CI = 8.5 – 8.9) years evaluated for the prevalence of malaria and their haematological parameters at different altitudes, 246 were positive for malaria. Leucopaenia and microcytosis were common in 23.3% (CI = 20.4 – 26.7%) and 15.2% (CI = 12.7 – 18%) of the children, respectively. A majority of the malaria cases were asymptomatic with mean Hb level within the normal range (Table 1).

**Table 1:** Baseline clinical characteristics of study population

**Variables**		**Mean**	**SD**	**Median**	**Interquartile range**		**N**
Age (years)		8.26	2.2	8.8	4	15	728
Age groups (years)	≤ 6	5.5	0.7	6	4	6	145
	7-10	8.7	1.1	9	7	10	443
	>10	11.8	1	11	11	15	140
Temperature (°C)		37.2	0.4	37.3	35.5	39.1	728
Haemoglobin (g/dl)		11.84	1.4	11.9	5.6	16.3	726
Parasitaemia/μl blood		2262.6	15578.8	230	18	220000	246
MCV (fl)		82.8	9.02	83.4	52.8	105.6	726
WBsC × 10^9^/L		6	2.2	5.9	1.2	17.8	725
**Variables**				**n**		%	
Sex		Male		348		47.8	
		Female		380		52.2	
Asymptomatic malaria				158		64.2	
Anaemia (Hb <11g/dl)				144		19.8	
Leucopaenia (WBC < .4.5 × 10^9^/L)				169		23.3	
Microcytosis				110		15.2	
Hypochromasia				389		53.7	

### Malaria parasite and altitude

The prevalence of malaria parasites in pupils varied with altitude. Children at low lands had a significantly higher (P <0.001) prevalence of malaria (60.6%, CI = 54.6–65.9%) than those in middle belt (29.1%, CI = 23.9–34.8) %] and highlands (7.7%, CI = 6.1–9.8%) as shown in Table 2. The geometric mean parasite density (GMPD) was higher in middle belt (475) and highlands (388) than lowlands (233). This difference was equally significant at P <0.05 (Table 2). Post hoc analysis revealed the difference in GMPDs between the middle belt and the low lands to be significant at P <0.01(P = 0.004, CI = 0.1 – 0.5).

**Table 2:** Malaria parasite prevalence and density at different altitudes

**Altitude**	**Number examined**	**Prevalence (%) of malaria (n)**	**Malaria parasitaemia**	
			**GMPD**	**Range**
Lowlands	302	60.6 (183)	233	18 - 220000
Middle belt	141	29.1 (41)	475	27 - 42350
Highlands	284	7.7 (22)	388	26 - 92880
Total	727	33.8 (246)	275	18- 220000
Level of significance	χ2 = 184.4		F = 4.963^§^	
	P < 0.001^c^		P = 0.008^a^	

^a^ Significant at P <0.05 level, ^c^ significant at P <0.001 level.

^§^ Log transformed parasitaemia was used in analysis.

### Fever

Out of the 728 pupils, 218 (29.9%) were febrile. The prevalence of malaria was significantly higher (χ2 = 5.929, P = 0.02) in children that presented with fever (40.4%, CI = 33.8–47.2%) when compared with the afebrile subjects (31%, CI = 27–35.2%. Altitude had no impact on the prevalence of fever. Although not significant, the GMPD was higher in pupils that presented with fever (285/μL blood) than in the afebrile (269 /μL blood).

### Malaria parasite and haematological indices

**Figure 1:**
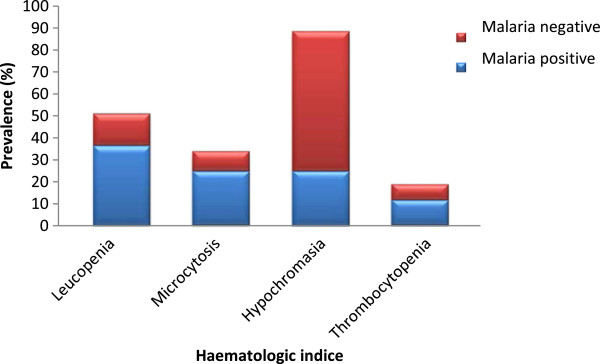
Prevalence of some haematologic indices by malaria status.

The prevalence of leucopaenia and microcytosis was significantly higher (P <0.001) in malaria parasitaemia-positive pupils (38.8%, CI = 32.6–45.2% and 26.5%, CI = 21.1–32.5%) than those negative (15.4%, CI = 12.4–19.1% and 9.4%, CI = 7–12.4%), respectively. In contrast, hypochromasia was significantly higher (P <0.001) in pupils who were malarial parasite negative (67.5%, CI = 63.1–71.6%) than those positive (26.5%, CI = 21.1–32.5%) as shown in Figure 1. Although not significant (P >0.05), pupils with AM had a higher prevalence of leucopaenia (43.7%, CI = 35.8–51.8%), microcytosis (27.2%, CI = 20.5–34.9%), hypochromasia (27.8%, CI = 21–35.5%) and thrombocytopaenia (14.9%, CI = 8.9–22.8%) when compared with those with CM (30.7%, CI = 21.3–41.4%; 25%, CI = 16.4-35.4%; 23.9% CI = 15.4–34.1% and 7.4%, CI = 2.4-16.3% respectively).

Overall, all mean haematological parameters were comparable in pupils with CM and AM but for the mean WBC counts. Pupils with AM had lower mean WBC counts (5.1 ± 2.5 × 10^9^/L) when compared with those with CM (5.9 ± 2.3 × 10^9^/L). The mean difference (0.78 × 10^9^/L) was statistically significant (t = 2.4, P =0.02) at 95% CI (0.14-1.4).

Children with AM had significantly lower (P <0.05) mean Hb, Hct, RBC, WBC, MCV and platelet counts than their negative counterparts (Table 3). Whilst MCH was comparable in children with AM and those negative, MCHC was significantly higher (P <001) in children with AM than those negative (Table 3).

**Table 3:** A comparison of mean haematological indices in asymptomatic malaria (AM) and negative (Neg) children

**Variable**	**Malaria status**	**N**	**Mean** (**SD**)	**t**- **test**, **P**-**value**	**95**% **CI of difference**
Hb (g/dL)	AM	158	11.4 (1.4)	−5.5	−0.8- - 0.85
	Neg	479	12.1 (1.2)	<0.001^c^	
Hct (%)	AM	158	34.2 (5.1)	−9.6	−5.5- -3.6
	Neg	479	38.8 (5.2)	<0.001^c^	
RBC × 10^12^/L	AM	158	4.4 (0.7)	−3.4	−0.3 - -0.1
	Neg	479	4.6 (0.5)	0.001^c^	
WBC × 10^9^/L	AM	158	5.1 (2.5)	−6.2	−1.5- -0.8
	Neg	478	6.3 (1.9)	<0.001^c^	
MCV (fl)	AM	158	78.4 (8.2)	−8.7	−8.3- -5.2
	Neg	479	85.2 (8.6)	<0.001^c^	
MCH (pg)	AM	158	26.5 (6.5)	0.03	−0.7- 0.7
	Neg	479	26.5 (2.5)	1	
MCHC (g/L)	AM	158	332.3 (22.5)	8.2	15.1- 24.8
	Neg	479	312.4 (27.9)	<0.001^c^	
Platelet × 10^9^/L	AM	114	294.3 (139.3)	−2	−72.3 – 1.3
	Neg	117	331.1 (134.1)	0.04^a^	

^a^ Significant at P <0.05 level, ^c^ significant at P <0.001 level.

The parasite density category did not have any significant effect on the mean Hct, WBC, RBC, platelet counts and MCH. On the other hand, while the mean Hb and MCHC significantly (P <0.05) decreased with an increase in parasite density category, a significant (P <0.01) increase was observed in MCV with parasite density category (Table 4).

**Table 4:** Mean red cell indices as affected by parasite density category

**Parasite density category**	**N**	**Hb (g/dL)**	**MCV (fl)**	**MCHC (g/L)**
		**Mean (SD)**	**Mean (SD)**	**Mean (SD)**
Low (<1,000)	211	11.5 (1.4)	78 (7.9)	334.6 (22.3)
95% CI of the means		11.3 – 11.7	77 – 79.1	331.6 – 337.7
Moderate (1,000-4,999)	24	10.8 (2.1)	79.3 (9.3)	327.1 (25.6)
95% CI of the means		9.9 – 11.7	75.3 – 83.2	316.3 – 337.8
High (5,000-99,999)	10	10.7 (1.5)	84.6 (7.8)	304.7 (31)
95% CI of the means		9.6 – 11.9	79.1 – 90.2	282.5 - 326
F		3.7	3.4	8.9
P		0.03^a^	0.04^a^	0.001^b^

^a^ Significant at P <0.05 level , ^b^ significant at P <0.01 level.

### Haematological indices, malaria parasite status and altitude

A statistically significant decrease (P <0.001) in prevalence of leucopaenia with altitude was observed irrespective of the status of malaria parasitaemia. On average the prevalence of leucopaenia in pupils in lowlands was 47.3% (CI = 42.1–53.6%), 8% (CI = 4.5–14.5%) in middle belt and 6.3% (CI = 2.7–8.2%) in highlands.

**Figure 2:**
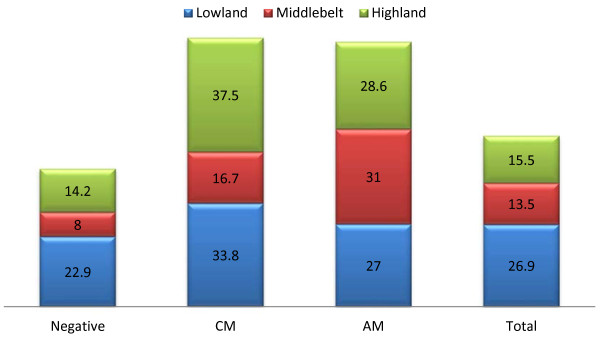
Prevalence of anaemia as affected by clinical status and altitude.

Overall, the prevalence of anaemia was significantly highest (χ2 = 16.4 P <0.001) in pupils in lowlands when compared with those in middle belt and highlands (Figure 2). A significant (χ2 = 10.3 P = 0.01) variation in the prevalence of anaemia with clinical status was observed in pupils in the middle belt. The prevalence of anaemia was highest in pupils with AM (31%, CI = 17.3– 49.2%) when compared with CM (16.7%, CI = 4.7–44.8%) and those negative (8%, CI = 4.1 – 15%) as shown in Figure 2.

In the lowland and highlands, the mean haematological values were comparable in children with CM and AM but for the mean WBC counts. The mean WBC count was significantly higher (P <0.05) in children with CM in the lowland than their asymptomatic counterparts (Table 5). On the contrary, a significant difference (P <0.05) in mean Hb, Hct, RBC and MCHC values with clinical status was observed in pupils in the middle belt. The mean Hb, Hct and RBCs were higher in children with CM when compared with those with AM. Conversely, the MCHC was significantly lower (P <0.05) in pupils with CM than those with AM (Table 5).

**Table 5:** Variation in mean haematological values as affected by clinical status and altitude

**Variable**	**Status**	**Lowlands**			**Middle belt**			**Highlands**		
		**N**	**Mean (SD)**	**P Value† 95% CI**	**N**	**Mean (SD)**	**P Value† 95% CI**	**N**	**Mean (SD)**	**P Value ‡**
Hb (g/dL)	CM	68	11.3 (1.7)	0.6	12	12.5 (1.4)	0.01^a^	8	11.4 (1.1)	0.9
	AM	115	11.4 (1.6)	-0.6 - -0.3	29	11.4 (1.1)	0.3-1.9	14	11.5 (1)	
Hct (%)	CM	68	32.5 (5)	0.5	12	42.2 (4.1)	<0.001^c^	8	38.1 (3.5)	0.8
	AM	115	33 (4.9)	-2-1	29	37.1 (3.9)	2.4-7.9	14	38.5 (3.9)	
RBC × 10^12^/L	CM	68	4.3 (0.7)	0.2	12	4.9 (0.4)	0.001^b^	8	4.4 (0.4)	0.8
	AM	115	4.4 (0.7)	-0.3-0.7	29	4.3 (0.6)	0.3-1	14	4.4 (0.5)	
WBC × 10^9^/L	CM	68	5.8 (2.5)	0.03^a^	12	6 (1.3)	0.9	8	7.1 (2.2)	0.07
	AM	115	4.9 (2.7)	0.1-1.7	29	5.9 (1.1)	-0.8-0.9	14	5.6 (1.5)	
MCV (fl)	CM	68	76.1 (6.4)	0.2	12	86.1 (7.6)	0.7	8	87 (7.7)	0.8
	AM	115	75 (6.4)	-0.8-3	29	86.8 (5.4)	-5-3.4	14	88.2 (4.8)	
MCH (pg)	CM	68	26.2 (1.9)	0.9	12	25.4 (2.8)	0.09	8	26.1 (2.4)	0.8
	AM	115	26.4 (7.5)	-2-1.7	29	26.9 (2.5)	-3.3-0.3	14	26.5 (2.2)	
MCHC (g/L)	CM	68	344.2 (16.9)	0.5	12	295.3 (22)	0.04^a^	8	300.1 (4.3)	0.8
	AM	115	342.7 (15.2)	-3.2-6.3	29	306.8 (12.5)	-22.5-0.5	14	300 (14.3)	

^a^ Significant at P <0.05 level , ^b^ Significant at P <0.01 level, ^c^ significant at P <0.001 level.

† Statistical significance determined by t-test.

‡ Statistical significance determined by Mann-Whitney U test.

### Confounding influences on haematological indices

Several exploratory MLR (enter) models were run with each haematological variable as the dependent variable to examine the influence of altitude, age, sex, parasitaemia, temperature, febrile status and SES on each haematologic variable. Overall, the sex, temperature and febrile status had no significant (P >0.05) influence on the haematological parameters under study. On the other hand, the age (P <0.001), altitude (P <0.001), SES (P = 0.04) and clinical malaria status (P = 0.001) significantly influenced the haematological parameters (N = 746, R^2^ = 0.4, P <0.001). In the 246 malaria parasitaemia positive children; altitude significantly (P <0.05) influenced the Hct, WBC, MCV and MCHC; age had an effect on the Hb, Hct, WBC and MCV; while parasitaemia influenced the Hb, Hct RBC and WBC counts (Table 6).

**Table 6:** Multiple linear regression analyses examining the influence of independent variables on each haematologic measure

**Haematological variable**	**Mean (SD)**	**Independent variable**	**β value**	**P value**	**R**^**2**^
Hb (g/dL)	11.4(1.5)	Altitude	0.1	0.4	0.3
		Age	0.2	0.001^b^	
		Parasitaemia	−0.2	0.01^a^	
Hct (%)	34.2 (5.4)	Altitude	0.4	<0.001^c^	0.3
		Age	0.2	<0.001^c^	
		Parasitaemia	−0.1	0.03 ^a^	
RBC × 10^12^/L	4.4 (0.7)	Altitude	0.04	0.5	0.1
		Age	0.1	0.05	
		Parasitaemia	−0.2	0.03 ^a^	
WBC × 10^9^/L	5.42 (2.5)	Altitude	0.2	0.01^a^	0.1
		Age	−0.2	<0.001^c^	
		Parasitaemia	0.1	0.02^a^	
MCV (fl)	78.4 (8.1)	Altitude	0.57	<0.001^c^	0.4
		Age	0.13	0.02^a^	
		Parasitaemia	0.02	0.07	
MCHC (g/L)	332.7 (23.7)	Altitude	−0.7	<0.001^c^	0.5
		Age	−0.01	0.05	
		Parasitaemia	−0.03	0.6	

^a^ Significant at P <0.05 level , ^b^ Significant at P <0.01 level, ^c^ significant at P <0.001 level.

N = 246.

## Discussion

A large proportion of children in malaria-endemic areas have parasites without clinical symptoms. The clinical consequences of asymptomatic malaria may vary across different epidemiological settings. This study examines the haematological hallmarks that may be associated with clinical and asymptomatic malaria in children at different altitudes in the Mount Cameroon region.

The overall malaria parasite prevalence observed in this study population was lower than the values reported by Kimbi *et al*[19], Nkuo-Akenji *et al*[8] and Kimbi *et al*[20] at various sites in the Mount Cameroon region. The decrease in the prevalence of malaria parasites may be due to the control measures recently implemented by the Cameroon Government through the Ministry of Public Health [21]. The measures include a nationwide free distribution of insecticide-treated bed nets (ITNs) as well as long-lasting insecticidal nets (LLINs) across the national territory. A decline in malaria burden attributed to the use of interventions such as ITNs and LLINs has also been reported in malaria-endemic countries such as Kenya [3] and Tanzania [22].

The decline in malaria parasite prevalence in this region may also be credited to a switch in the treatment of malaria with sulphadoxine-pyrimethamine due to widespread resistance to more effective artemisinin-based combination therapies as first-line treatment since 2004 in Cameroon [23]. Furthermore, following the change in treatment policy, the National Malaria Control Programme organized workshops across all regions in Cameroon to educate and inform health workers at public, mission and private health facilities of the change in policy and the need to adhere to it. These educative talks as well as the free treatment of children under five, as decreed by the Head of State, may have contributed to the decline in the burden of malaria.

Fever and history of fever are fairly sensitive measures of malaria and infection with *P*. *falciparum* is very likely to result in symptoms that include fever. However, fever alone remains a poor discriminator of malaria infection in tropical Africa where children are likely to have manifold infections with several other pathogens. Nonetheless, in line with reports by Kimbi *et al*[19] and Achidi *et al*[7] in the same region, findings from the study revealed a significant association between fever and the presence of malaria parasite. However, the majority (64.2%) of malaria parasite-positive pupils were asymptomatic, indicating they may have developed an anti-disease immunity, but anti-parasite immunity has not reached levels high enough to clear the infection [24].

On the other hand, a good number of febrile subjects (59.6%) were malaria parasite negative. The high temperatures in these pupils could be attributed to other infections, such as co-infections with other parasites, viruses or bacteria, which were beyond the scope of this study. Nonetheless, this is a cause for concern because presumptive treatment for malaria with fever as a marker is the order of the day amongst most parents in the Mount Cameroon region. These parents administer treatment themselves at home and only seek medical attention when the fever persists [8]. It is therefore imperative, as stated by Okiro and Snow [3], that all fevers should be tested to corroborate or contest the role of malaria in the febrile presentation.

In line with Achidi *et al*[7] malaria parasite prevalence was higher in children living at lower altitudes than their higher altitude counterparts. The decrease in prevalence of malaria with an increase in altitude could be credited to favourable environmental and climatic conditions in the lower altitude, which promote the rapid growth of the anopheline vectors and consequently a high rate of malaria transmission. Further observations from the study revealed pupils in middle belt and highlands had higher GMPD than those in lowlands. On the contrary, several studies [22,25] reported intense parasitaemia in children living at low altitudes when compared with their high-altitude counterparts. The lower parasitaemia observed in pupils at lower altitude could be attributed to the fact that children in high malaria transmission areas are continuously exposed to infection and quickly acquire and maintain protective immunity against severe disease, so that during subsequent episodes they may suffer less severe forms of uncomplicated malaria [25]. Perennial, intense malaria transmission however results in considerable degree of immunity after early childhood [26].

It is worth noting that the pupils in this study were seemingly healthy, with regular school attendance, even though a pupil with temperature as high as 39.1°C was recorded. Also, the majority (64.2%) of the pupils positive for malaria parasites were asymptomatic and remarkably the length of period they were parasitaemic unclear. This may have contributed to the higher prevalence of leucopaenia, microcytosis and thrombocytopaenia observed in children with AM when compared with those with CM and those negatives. This highlights the insidious effects AM may have on the haematological components of children living in an area of intense malaria transmission.

The presence of malaria parasites in these pupils was found to be associated with lower Hb, Hct, RBC and WBC counts as confirmed by some of the negative beta values in the models. The higher prevalence of AM than CM most likely would have exacerbated the reduction in the red cell indices as asymptomatic parasitaemia and protracted malaria infections have been associated with a marked reduction in Hb concentration [27] and with a clinically significant RBC destruction [28].

Even though *Plasmodium* infection is thought to lower erythrocyte counts and Hb levels by inducing haemolysis, decreased erythropoiesis and increased clearance of both parasitized and non-infected erythrocytes, findings from the study revealed no significant effect of the parasite density on the mean RBC count. The significant decrease in mean Hb levels with an increase in parasite density without a corresponding significant decrease in RBC count may be related to the extraction of *P*. *falciparum* parasites from RBCs, leaving the RBCs intact. Kumaratilake *et al*[29] reported that human neutrophils *in vitro* are capable of extracting *P*. *falciparum* parasites from RBCs, leaving the RBCs intact and the removal of intra-erythrocytic parasites occurs naturally *in vivo* when the host immune system can act. Also, parasitized RBCs treated with anti-malarial drugs have been recorded extruding dead trophozoites [30].

Further observations from the study revealed the altitude not to be of any significance on the Hb levels and RBC counts, rather these haematological measures were influenced mostly by the age and parasite density. Majority (184/246) of the pupils with malaria parasitaemia were above seven years of age with 87.5% (161/184) of them having parasite density <1000/μl of blood. The age related decrease in malaria parasite density observed is probably related to the acquisition of protective immunity due to repeated infections as children grow older in high transmission areas. This protective immunity may have permitted the host immune system to act consequently the non-significant decrease in mean RBC counts.

Reports on the impact of *Plasmodium* infection on WBC counts are conflicting. Consistent with previous investigations [7,9,11,31,32] a decrease in WBC counts was associated with malaria infection while Ladhani *et al*[33] reported children with malaria to have a higher WBC count. The mean WBC counts in the pupils highlights a significant interaction between the age, parasite density and the altitude. The mean WBC count increased with altitude, decreased with age and was relatively low in pupils with malaria parasite counts less than 1000/μL of blood. The relatively low leucocyte counts in pupils in lower altitude when compared with the other altitudes may be ascribed to the high prevalence of malaria parasitaemia in the pupils, majority (91.2%) of whose parasite counts were in the lowest category. The high prevalence of leucopaenia and the significantly low mean WBC count observed in pupils with AM may well be protective against severe disease. Further studies on the role of leucopaenia in asymptomatic malaria are required to determine the significance of these findings.

Findings from the study revealed that thrombocytopaenia is common among pupils with malaria parasitaemia and a significant (β = 03, P <0.001) relationship exists between the platelet counts and the WBC counts. The prevalence of thrombocytopaenia (14.9%) in pupils with AM in the study population is comparable to those obtained by Ladhani *et al*[33] in a similar group of children. Thrombocytopaenia in malaria infection is likely to be due to excessive splenic pooling of the platelets and decreased platelet lifespan [34], which in turn, may be a consequence of immune-mediated platelet destruction [35].

An evaluation of the effect of parasite density category on MCV showed an increase in MCV with an increase in parasite density category. Parasitized red cells are known to develop knobs, which facilitate their adhesion to endothelial cells of capillaries [36], hence non-parasitized red cells form the bulk of circulating red cells before destruction. The increase in MCV may be the result of the chemical and physical change effects on the non-parasitized erythrocytes [37] which spend more time in circulation than the parasitized erythrocytes.

The decrease in prevalence of anaemia with increase in altitude observed in the study is not unexpected as most red cell indices have a positive relationship with altitude as indicated by their β value. The level of Hb (<11 g/dL) used as an indicator of anaemia was not significantly influenced by the altitude. However, there were similarities in trend in the prevalence of anaemia and malaria parasite prevalence with altitude. Furthermore, pupils in the middle belt had the highest GMPD with significant variations in the mean haematological values when compared with the other altitudes. Higher parasite intensity may lead to lower mean Hb levels and consequently anaemia although the density of malaria infection is not a significant factor in determining disease severity [38].

The significant differences in haematological parameters with clinical status observed in children in the middle belt (Table 5) may be attributed to a delay in acquired immunity with increasing altitude. The negative impact of parasite density and the reduction in transmission intensities with altitude may have resulted in a delay in acquired anti-disease immunity that depends on the frequency of parasite exposure since birth. However, the observations point out the underlying effects of asymptomatic malaria on haematological indices.

## Conclusions

With a decline in the burden of malaria in school-aged pupils, there is a need for differential diagnosis of all fevers to corroborate the role of malaria in the febrile presentation. Altitude influenced the distribution and density of malaria parasites and was of confounding influence on the haematologic profiles. The results highlight the insidious effects of AM on the haematologic components. However, further studies to assert the protective role of leucopaenia in asymptomatic malaria is necessary.

## Abbreviations

AM: Asymptomatic malaria; CM: Clinical malaria; CI: 95% confidence interval; GMPD: Geometric mean parasite density; Hb: Haemoglobin; Hct: Haematocrit; ITNs: Insecticide-treated bed nets; LLINs: Long-lasting insecticidal nets; MCH: Mean corpuscular haemoglobin; MCHC: Mean corpuscular haemoglobin concentration; MCV: Mean corpuscular volume; MLR: Multiple linear regression; RBC: Red blood cell; SES: Socio economic status; WBC: White blood cell.

## Competing interests

The authors declare that they have no competing interests.

## Authors’ contributions

HKK conceived the study, participated in the design and co-ordination, data collection and revision of the manuscript; IUNS conceived and participated in the design, data analysis, interpretation, and write-up of the manuscript; MN participated in the collection of data and laboratory analysis; JKAK participated in supervision and revision of the manuscript; EL participated in the collection of data and laboratory analysis; YN participated in data collection, LMN participated in coordination and revision of manuscript; HN carried out laboratory analysis; LGL participated in co-ordination and revision of manuscript. All authors read and approved the final manuscript.
